# Gastrointestinal, Behaviour and Anxiety Outcomes in Autistic Children Following an Open Label, Randomised Pilot Study of Synbiotics vs Synbiotics and Gut-Directed Hypnotherapy

**DOI:** 10.1007/s10803-024-06588-9

**Published:** 2024-10-17

**Authors:** Leanne K. Mitchell, Helen S. Heussler, Christopher J. Burgess, Ateequr Rehman, Robert E. Steinert, Peter S.W. Davies

**Affiliations:** 1 Child Health Research Centre, Faculty of Medicine, The University of Queensland, South Brisbane, QLD Australia; 2 Child Development Program, Children’s Health Queensland, Brisbane, QLD Australia; 3 Centre for Clinical Trials in Rare Neuro Developmental Disorders, Children’s Health Queensland, Brisbane, QLD Australia; 4 Department of Gastroenterology, Hepatology and Liver Transplant, Queensland Children’s Hospital, Brisbane, QLD Australia; 5 DSM-Firmenich, Health, Nutrition & Care (HNC), Kaiseraugst, Switzerland; 6 Department of Surgery, Division of Visceral and Transplantation Surgery, University Hospital Zurich, Zurich, Switzerland

**Keywords:** Autism, Asperger’s, Functional gastrointestinal disorders, Gut disorders, Gut-brain

## Abstract

Alterations of the microbiome-gut-brain (MGB) axis have been associated with autism spectrum disorder (ASD) and disorders of gut-brain interaction (DGBI). DGBI are highly prevalent in autistic children and are associated with worsening behaviour and anxiety. Treatments such as probiotics, prebiotics and gut-directed hypnotherapy (GDH) have shown efficacy in improving gut symptoms in children. The primary objective of the study was to compare changes in gastrointestinal (GI) scores following a 12-week intervention of synbiotics (prebiotic + probiotic) +/- GDH with a follow-up at 24 weeks. Secondary objectives included changes in behavioural and anxiety symptoms, while changes in gut microbiome composition were assessed as an exploratory objective. Children diagnosed with ASD aged 5.00-10.99 years (*n* = 40) were recruited and randomised (1:1) to a 12-week intervention of either synbiotics (SYN group) or synbiotics + GDH (COM group). Both the SYN and COM group experienced significant reductions in total GI scores post-intervention and at follow-up (*p* < 0.001), with no superiority of the COM treatment over the SYN treatment. The COM group showed beneficial reductions in anxiety scores (*p* = 0.002) and irritability behaviours (*p* < 0.001) which were not present in the SYN group. At follow-up, only those in the COM group maintained significant reductions in GI pain scores (*p* < 0.001). There were significant changes in gut microbiota such as increases in *Bifidobacterium animalis* and *Dialister* in both groups over time. In conclusion, synbiotics with or without GDH may help support standard care for autistic children who suffer comorbid DGBI. The trial was prospectively registered at clinicialtrials.gov on 16 November 2020 (NCTO4639141).

**Supplementary Information:**

The online version contains supplementary material available at 10.1007/s10803-024-06588-9.

Autistic children commonly experience disorders of gut-brain interaction (DGBI, formerly known as functional gastrointestinal disorders) such as constipation, diarrhea, bloating/flatulence and abdominal pain (Leader et al., 2022; Penzol et al., 2019). Comorbid gastrointestinal (GI) symptoms are positively associated with anxiety (Mazurek et al., 2013) and autism severity levels (Karagözlü et al., 2021; Yang et al., 2018), and yet standard therapy for autistic children fails to address these comorbidities. The high prevalence of DGBI in autism adds to the emerging hypothesis that alterations of the microbiome-gut-brain (MGB) axis may play a role in autism spectrum disorder (ASD) (Davies et al., 2021). However, the cause of microbial change in ASD is still largely debated. Recent evidence suggests microbial alternations may be due to ASD-associated restrictive eating behaviours (Yap et al., 2021), while other research suggests changes may be evident in early infancy before ASD diagnosis (Pärtty et al., 2015). Regardless of the cause, treatment aimed at modulating the GI microbiome may prove beneficial for individuals with ASD. In support of this, Li and colleagues (Li et al., 2022) found that modulating the GI microbiome via faecal microbial transplant (FMT) alleviated behavioural and GI symptoms in autistic children. While FMT offers a novel potential treatment in ASD, finding less invasive and easily accessible options for autistic individuals should also be explored.

Preliminary evidence suggests a role for pre- and probiotic supplements in managing both GI and behavioural symptoms in autistic children (Mitchell & Davies, 2021; Thangaleela et al., 2022). Clinical probiotic studies in autistic children are heterogeneous, however, the most frequently trialled probiotic species (in combination or singularly) are *Lactobacillus acidophilus (L. acidophilus)*, *Lactobacillus plantarum (L. plantarum)*, *Lactobacillus rhamnosus GG (L. rhamnosus GG) and B. longum* (Mitchell & Davies, 2021). Independently these probiotics have shown efficacy in improving GI symptoms and intestinal barrier function (*L. plantarum WCFS1 and L. rhamnosus GG*) (Parracho et al., 2010; Francavilla et al., 2010), shifting ASD-associated microbial metabolites (*L. plantarum WCFS1* and *L. Acidophilus* Rosell-11) (Parracho et al., 2010; Kałużna-Czaplińska et al., 2012) and reducing anxiety-like behaviour (*B. longum*, animal trial) (Bercik et al., 2011). Additionally, the prebiotic fibre, partially hydrolysed guar gum (PHGG), is a well-tolerated supplement (Parisi et al., 2002) that has shown efficacy in improving GI symptoms in individuals with DGBI (Niv et al., 2016; Romano et al., 2013) and, in a study with autistic children, PHGG supplementation led to improved behavioural and gut outcomes (Inoue et al., 2019).Moreover, a psychotherapeutic approach known as gut-directed hypnotherapy (GDH) has shown efficacy in reducing GI symptoms in adults (Peters et al., 2016; Vasant & Whorwell, 2019) and children (Gulewitsch & Schlarb, 2017; Rutten et al., 2013) with DGBI. To the best of the authors’ knowledge, no previous research has assessed the utility of GDH in autistic individuals. GDH broadly aims to reduce anxiety and normalise gut-based interoception using relaxation techniques, guided imagery and suggestive storytelling and metaphors (Vasant & Whorwell, 2019).

This study aimed to assess the effectiveness of a pre- and probiotic supplement (i.e. synbiotic, SYN group) versus a synbiotic supplement combined with GDH (COM group) on GI symptoms in autistic children. The study synbiotic contained 5g of partially hydrolysed guar gum (PHGG) and a probiotic mixture of Humiome^®^ *L. rhamnosus* GG (ATCC 53103), Humiome^®^ *L. plantarum* DSM 34532 (formerly known as L. plantarum TIFN101), *B. lactis* DSM 32269 and *B. longum* DSM 32946 (input ratio of 13.3 : 3.6 : 8.0 : 1). Secondary outcomes assessed changes in behaviour and anxiety, as well as an exploratory analysis of shifts in the stool microbiota post-intervention. Given the bi-directional nature of the MGB axis, it is hypothesised that the COM intervention will be more effective than the SYN intervention alone.

## Methods

### Study Design

**Fig. 1:**
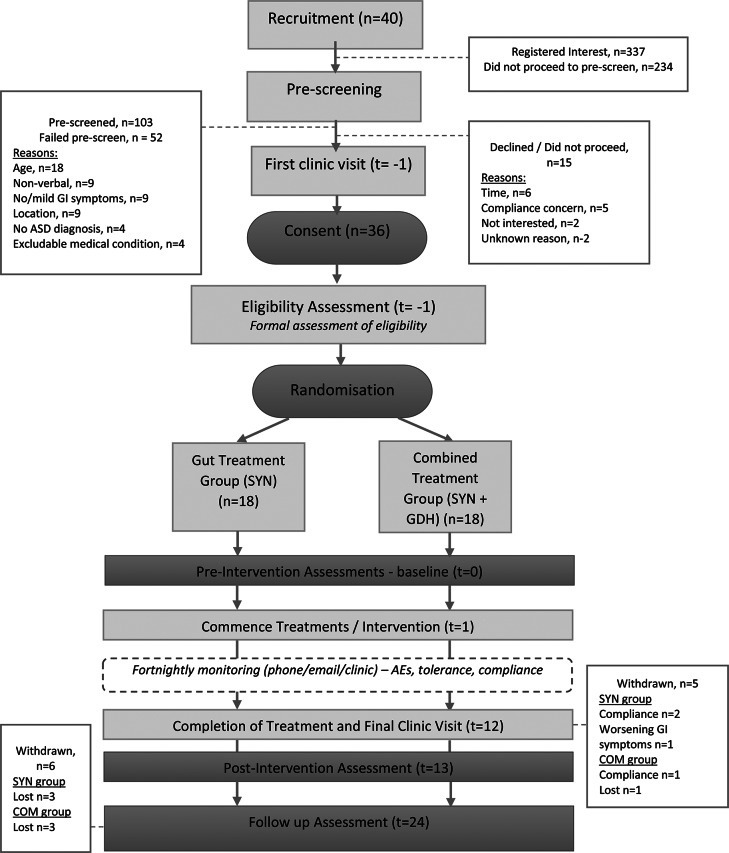
Study design and participant flowchart

The clinical trial was conducted in Brisbane, Australia (December 2020 to November 2021). It was an open label, randomised pilot study using block randomisation to maintain balance between treatments (Fig. 1). The allocation was determined by opening sequentially numbered envelopes containing the group to which the participant was assigned. The random allocation sequence was created using www.sealedenvelope.com by PSWD. Subjects were enrolled and assigned to their group only by LKM. The trial was not blinded and had no control group. Ethical approval was obtained from the Queensland Children’s Hospital and Health Service Human Research Ethics Committee (reference number HREC/20/QCHQ/58792) and ratified by the University of Queensland Human Research Ethics Committee (reference number 2020001211). The trial was prospectively registered at clinicialtrials.gov (NCTO4639141). Written informed consent was obtained from parents/guardians. Researchers supplied the study treatments at no cost to the parent/guardian or child participant. Reporting of safety events/AEs was in accordance with good clinical practice (GCP) as set out in the National Health and Medical Research Council’s ‘Safety monitoring and reporting in clinical trials involving therapeutic goods’ (National Health and Medical Research Council, 2016). Participants were monitored every two weeks from enrolment for AEs, compliance and any changes to their standard care. The de-identified log of AEs was revised by an independent safety monitor every two weeks from study commencement.

### Recruitment

Potential participants were sourced from the community through ethically approved study flyers posted on autism-associated social media sources and through non-for-profit organisations. Children were eligible to enrol if they were between 5.00 and 10.99 years old and had a confirmed diagnosis of ASD or Pervasive Development Disorders (PDD) including autistic disorder, Asperger’s disorder, PDD not otherwise specified and atypical autism. Children were also required to have a diagnosed DGBI or a score of three or more on the six-item gastrointestinal severity index as assessed at the screening visit (6-GSI) (Adams et al., 2011). Children were ineligible if they were non-verbal or had any of the following conditions: an organic GI disorder (Inflammatory Bowel Disease, Coeliac Disease or current GI tract infection); immunocompromised or severely ill; bipolar, schizophrenia, or personality disorder; diabetes mellitus; or a diagnosed eating disorder. Selection criteria were assessed at the screening visit based on parental report. A copy of the child’s letter of diagnosis (given by a qualified professional) against the Diagnostic and Statistical Manual of Mental Disorders (DSM) criteria was required as evidence of ASD/PDD diagnosis before enrolment. DGBI were confirmed through obtaining a copy of a letter or email from the child’s treating doctor. Potential participants required a 1-month washout period following antibiotic/antifungal use and a two-week washout period following probiotic/prebiotic supplements (Adams et al., 2011).

### Study Intervention

Participants were randomized (1:1) to either the synbiotic treatment group (SYN group) or the combined treatment group (synbiotic + GDH) (COM group). The intervention period was 12 weeks (week 12, t = 12), with an additional 12-week follow-up (week 24, t = 24). Participants continued their standard care (e.g. therapies, medication, diet) throughout their enrolment and parents/guardians were asked to avoid making any changes unless medically indicated. Changes to standard care were reported and assessed during the fortnightly (every 2 weeks) monitoring process.

Participants randomised to receive the synbiotic were required to consume one sachet per day which contained 5 g of partially hydrolysed guar gum (PHGG) and a probiotic mixture containing Humiome^®^ *L. rhamnosus* GG (ATCC 53103), Humiome^®^* L. plantarum* DSM 34532 (formerly known as L. plantarum TIFN101), *B. lactis* DSM 32269 and *B. longum* DSM 32946 at an input ratio of 13.3 : 3.6 : 8.0 : 1. The initial total cell count was 3.8 × 10^10^ colony forming units (CFU) at study start and 2.5 × 10^9^ CFU at study end. Instructions were provided to mix the synbiotic in a chilled or room temperature liquid drink or semi-liquid food item. During the first week of the trial, parents/guardians were instructed to administer half a sachet only, to help improve tolerability.

Children randomised to receive the combined treatment (synbiotic + GDH) were required to take the daily synbiotic (as above) together with daily use of the GDH home-based therapy program. The home therapy program consisted of six therapy sessions/recordings over 12 weeks. Each recording (sessions 1 through 6) were approximately 15 min in duration and used in succession, daily for a period of two (2) weeks (14 days). The program was based on the work of Vlieger et al. (Vlieger et al., 2007) using the evidenced-based Manchester model of GDH (Gonsalkorale, 2006). The program was further adapted for autistic children by the Principal Investigator (a DGBI dietitian and a clinical hypnotherapist) in consultation with a behavioural paediatrician who utilises GDH in clinical practice. The GDH protocol aims to reduce anxiety and normalise gut-brain interoception. The broad themes of each session were as follows: Session 1 - introduction to techniques + ego strengthening; Session 2 – relaxation; Session 3 – safety/control and colon visualisation; Session 4 – self-healing, control and normalisation of GI tract function; Session 5 – anxiety/stress reduction; Session 6 – control and normalisation of GI tract. The session recordings followed a fixed structure and scripting, however, were overlaid with personalised examples and verbiage (for example, favourite colour/animal/place and the words [or the opposite terms] the participant used to describe their GI issues). This information was gathered at enrolment.

### Objectives

The primary objective of this trial was to compare changes in GI severity scores as measured by the 6-GSI from baseline (week 0, t = 0) to post-intervention (week 12, t = 12) and at follow-up (week 24, t = 24) within and between treatment groups (SYN group vs. COM group). Stool consistency was also assessed using the Bristol Stool Chart (BSC). The secondary objectives included changes to behavioural scores as measured by the Aberrant Behaviour Checklist (ABC) and anxiety symptoms as measured by the Parent-Rated Anxiety Scale – ASD (PRAS-ASD). Shifts in gastrointestinal microbiome were assessed using stool metagenomic DNA sequencing as an exploratory objective of this study.

### Measurement Tools

#### Six-item Gastrointestinal Severity Index (6-GSI)

GI symptom severity was assessed using a modified version of the Gastrointestinal Severity Index (GSI) (Schneider et al., 2006). The GSI was modified to a 6-item tool by Adams et al., (Adams et al., 2011) and has been used successfully in subsequent research investigating GI symptoms in autistic children (Shaaban et al., 2018). This parent-rated tool measures six items of GI dysfunction - constipation, diarrhea, stool consistency, stool smell, flatulence and abdominal pain, producing a total GI severity score. The 6-GSI assesses each GI symptom using a Likert scale of 0–2 (0 = nil/mild/infrequent; 1 = moderate/occasional; 2 = severe/frequent). Mild GI issues are defined as a total score of < 3 and moderate or severe GI issues are defined as a score of ≥ 3 (Shaaban et al., 2018). A reduction of 2-points on this tool over the intervention period was considered clinically significant. A 2-point reduction equates to an improvement in one GI symptom from “severe” to “mild” or a change across two GI symptoms from “severe” to “moderate” or “moderate” to “mild”.

#### Bristol Stool Chart (BSC)

Stool consistency was measured using the BSC. The BSC is a widely used tool to help individuals assess their bowel motions based on a visual scale of 1–7 stool types (Type 1–3 = hard stool indicative of constipation; Type 4 = normal; Type 5–7 = loose stool indicative of diarrhoea) (Lewis & Heaton, 1997). A child-friendly version of the BSC chart was provided and assessment was made by the parent/guardian (visual inspection) and/or child participant (if toileting independently). The BSC is effective at measuring changes in colonic transit with evidence of utility in both clinical and research settings (Lewis & Heaton, 1997).

#### The Aberrant Behaviour Checklist – Community – Version 2 (ABC)

Behavioural changes were assessed using the ABC (Aman & Singh, 1986). The ABC was developed to assess treatment effectiveness and has been extensively used in paediatrics research due to its high reliability and validity (Schmidt et al., 2013). The factor structure and psychometric properties of the ABC have been validated among a large sample of autistic children (*n* = 1,893) and normative data is available for this cohort (Kaat et al., 2014). The tool is a 58-item checklist scored on a scale of 0–3, with higher scores indicating greater severity or behavioural difficulties. It consists of five subscales: Irritability (ABC-I = 15 items); Social Withdrawal (ABC-SW = 16 items); Stereotypic Behaviour, (ABC-SB = 7 items); Hyperactivity/Non-compliance (ABC-HNC = 16 items) and Inappropriate Speech, ABC-IS = 7 items). The authors of the ABC state that 0.5 SD reduction in scores from pre- to post-intervention is clinically relevant (Aman & Singh, 2017). SD values for this cohort can be sourced from normative data produced by Kaat et al., 2014 (Kaat et al., 2014). Using the normative data from this study, 0.5 SD values can be calculated for 6–12-year-old autistic children as follows: ABC-I = 4.65; ABC-SW = 3.55; ABC-SB = 2.10; ABC-HNC = 5.45; and ABC-IS = 1.45. This data will be used when reporting the results to gauge the clinical significance. The ABC-I is the mostly widely used behavioural subscale to measure treatment effectiveness (especially in pharmaceutical studies) (Aman, 2013) and the key subscale of interest for behavioural outcomes in this study.

#### The Parent-Reported Anxiety Scale – Autism Spectrum Disorder (PRAS-ASD)

Anxiety symptoms were assessed using the PRAS-ASD (Scahill et al., 2019). This tool was developed for use in autistic children aged 5 to 17 years old and has demonstrated good validity and reliability across an online (*n* = 990) and clinical (*n* = 116) cohort (Scahill et al., 2019). The tool is a 25-item questionnaire. Each item is scored as 0 (none), 1 (mild), 2 (moderate), or 3 (severe). The sum provides an overall total (maximum score is 75) with a higher score indicating higher anxiety levels.

#### Stool Microbiota

Shifts in microbiota composition were analysed using stool metagenomic DNA sequencing performed by Microba Life Sciences. Participants used a home collection kit to collect stool samples at baseline and post-intervention (week 8). Stool samples were collected using a Copan FLOQSwab (Copan, USA) that has an active drying system to stabilise the sample. Samples were returned to Microba Life Sciences laboratories via express postal packs. On arrival at the laboratory, samples were visually inspected for quality control (QC) issues, stored at -20 °C and collated into batches for processing.

DNA extraction was performed using the DNeasy 96 PowerSoil Pro QIAcube HT Kit (Qiagen 47021), with proprietary workflow optimization steps on the QIAcube HT DNA extraction system (Qiagen 9001793). Libraries were prepared using a modified protocol, using Illumina^®^ DNA Prep, (M) Tagmentation (96 Samples) kit (Illumina #20018705). The libraries were pooled at equimolar amounts. The resulting sequencing pool was quantified, and QC was performed with gel analysis, qubit measurement and qPCR. The library was prepared for sequencing (Illumina) using a NovaSeq6000, v1.5 reagents and 2 × 150 bp paired-end chemistry. Pools were sequenced to a standard depth of 3Gb per sample unless otherwise agreed upon (approximately 7 M – 16 M paired-end reads). Microbial data generated from the metagenomic sequencing of the study samples was processed using key proprietary bioinformatic systems (Microba Genome Database (MGDB) and Microba Community Profiler (MCP) (Parks et al., 2021).

### Statistical Methods

The sample size was powered on the primary outcome using previously published data (Adams et al., 2011). A sample size of 16 participants in each arm was required to have 80% power at 5% significance (2-sided) to detect a one Standard Deviation (SD) difference between groups. A 25% margin was added to the sample size to allow for potential drop-outs and missing data. This resulted in a target of 20 participants in each arm, giving a total sample size of 40 participants.

Statistical analysis for the primary and secondary objectives was performed using the Statistical Package for Social Sciences (SPSS) program (IBM SPSS Statistics for Windows, Version 29.0). Results were considered statistically significant at P values < 0.05. Demographic and baseline characteristics were summarised by treatment group using descriptive statistics. Continuous baseline variables were described using means and standard deviations. Categorical variables are expressed as frequencies and percentages. The success of the randomisation process was analysed against key variables using independent t-tests (continuous variables) and chi-squared tests of association (categorical variables).

Unless otherwise stated data are shown as adjusted mean +/- standard error, due to the adjustments made to attempt to control for potential confounders (antibiotic exposure during the trial or unsuccessful randomisation of key baseline characteristics). Owing to the loss of participants from post-intervention (t = 12) to follow-up (t = 24), the adjusted mean scores varied between the analysis of the two timepoint (pre-intervention to post-intervention) and the three timepoint model (pre-intervention, post-intervention and follow-up).

Analysis was based on the modified intention-to-treat (mITT) population. This included all participants randomised to a treatment group who had valid/complete pre- and post-intervention measurements for the primary objective. Primary and secondary outcomes were assessed using two-way mixed ANOVAs to analyse the two-way interaction of time x treatment group. The assumptions for a two-repeat ANOVA were met for all outcome variables with the exception of ABC-SB and ABC-IS in the SYN group only. In the SYN group, outliers were evident for the pre- and post-intervention ABC-SB scores and for the pre-intervention ABC-IS scores. These were true values for which re-coding was not appropriate. Furthermore, as ABC-SB and ABC-IS are not the key subscales of interest for this study, adjustments to address these outliners was not deemed warranted. There were no outliers for any other outcome variable as assessed by boxplot. The assumption of normality was satisfied for all variables as assessed by visual inspection of normal Q-Q plots. There was homogeneity of variances (*p* > 0.05), and covariances (*p* > 0.05), as assessed by Levene’s test of homogeneity of variances and Box’s M test, respectively, for all outcome variables.

Analysis of all variables also included the use of dependent t-tests to assess the pairwise comparison (within group) effect of any treatment. Primary outcome data were also investigated according to response (responders vs. non-responders). Responders were classified as any participant who experienced a 2-point or greater reduction in gastrointestinal severity scores post-intervention. The response level was determined based on clinical significance (see *Measurement Tools*).

A secondary analysis of the dataset was undertaken on a per-protocol (PP) bases. PP inclusion was based on the level of treatment compliance as reported by parents/guardians. Participants were considered compliant if they consumed 90% of the synbiotic doses and, for those allocated to the combined treatment, listened to 50% of the home-therapy GDH program.

Exploratory and statistical analysis of microbial community profiles were performed in R version 4.1 or higher (R Core Team, 2019) using Microba Life Science’s MPR package version 2.1.0. Alpha diversity is a summary of community structure within a sample and was calculated using rarefied count data using two measures, the *Shannon Diversity index* (Spellerberg & Fedor, 2003) and *Richness*. Within-group changes at species, genus and family levels from baseline to post-intervention, were assessed by analysing the differentially abundant bacterial taxa using LMER (linear mixed effect regression) of centered log-ratio (clr) transformed relative abundances. Analysis was limited to a filtered set of features (e.g. microbial species). Low abundance (a maximum sample count of less than 100 for taxonomic data) and rare features (present in less than 3 samples) were excluded from the analysis. Results were considered statistically significant at P values < 0.05. P values are uncorrected given the effect of interventions on gut microbiome composition was an exploratory objective. The magnitude (median value at the end of intervention minus median values at baseline) and direction of difference are shown by delta values.

## Results

A total of 36 children were randomly assigned to the trial, including 18 in the SYN group and 18 in the COM group. Figure 1 shows the flowchart of participants throughout the study.

Five participants were excluded from the mITT analysis due to incomplete data for the primary outcome post-intervention (t = 12) and an additional six were excluded at follow-up (t = 24). The sole reason for incomplete data was withdrawal from the study, with three parents withdrawing their child due to non-compliance and one due to worsening GI symptoms. The remaining two participants excluded from the post-intervention (t = 12) analysis and the six excluded at follow-up (t = 24) were lost to follow-up. Therefore, the final analysis was based on 31 participants (SYN group, *n* = 15; COM group, *n* = 16) post-intervention (t = 12) and 25 participants (SYN group, *n* = 12, COM group, *n* = 13) at follow-up (t = 24). For details on withdrawn/excluded participants please refer to Fig. 1. During the study, no parents/guardians reported changing their child’s standard care in terms of therapies or diet (as reported in the fortnightly monitoring process). While four participants in the COM group required antibiotics during their enrolment.

No statistically significant differences between the treatment groups were found at baseline, except for sex and pre-intervention anxiety scores (see Table 1). The mean (SD) age of the children was 7.75 years (1.87), and the anthropometric data indicated that enrolled participants were on average heavier and taller than the reference data (21). Of the 31 participants, 18 were male (58.1%) of whom 11 were in the SYN group (73.3%) and 7 were in the COM group (43.8%). The most prominent ASD diagnosis was ASD level II (80.6%, *n* = 25) and parents reported high levels of “fussy” eating among enrolled children (90.3%, *n* = 28). The majority of participants had anxiety (83.9%, *n* = 26) and many had comorbid ADHD (61.3%, *n* = 19). At baseline the mean total 6-GSI score was 5.23 (SD 1.67) and the majority tended to experience “hard” stools (60.0% [*n* = 18] with stool types 1–3 on BSC).

**Table 1:** Demographic and baseline data

Baseline Characteristics	mITT population (*n* = 31)	Between Groups		
		mITT Synbiotic *(n = 15)*	mITT Combined *(n = 16)*	*p*-value^
Age in years (SD)	7.75 (1.87)	7.07 (1.86)	8.39 (1.70)	0.051
Gender – male (%)	18 (58.1)	11 (73.3)	7 (43.8)	0.045*
**Diagnosis (%)**				
ASD I	5 (16.1)	3 (20.0)	2 (12.5)	0.450
ASD II	25 (80.6)	12 (80.0)	13 (81.3)	
ASD III	1 (3.2)	0 (0.0)	1 (6.3)	
**Anthropometric Data – Mean (SD)**				
BMI z-score	0.45 (1.29) Missing = 3	0.15 (1.37) Missing = 2	0.70 (1.20) Missing = 1	0.268
Height z-score	0.40 (1.29) Missing = 3	0.34 (1.21) Missing = 2	0.44 (1.40) Missing = 1	0.849
Weight z-score	0.51 (1.35) Missing = 3	0.33 (1.25) Missing = 2	0.66 (1.46) Missing = 1	0.533
**Early Life Factors – Frequency (%)**				
Full term gestation	28 (90.3)	14 (93.3)	14 (87.5)	1.00
Vaginal birth	14 (45.2)	8 (53.3)	6 (37.5)	0.376
Infant Feeding				
Exclusive breastfeeding for 6 months	14 (45.2)	9 (60.0)	5 (31.3)	0.266
Exclusive breastfeeding less than 6 months	9 (29.0)	3 (20.0)	6 (66.7)	
Formula or mixed feeding from birth	8 (25.8)	3 (20.0)	5 (31.3)	
Antibiotic Exposure in the first 2 years of life				
None/not sure	7 (23.3)	3 (20.0)	4 (25.0)	0.200
1–4 times	16 (53.3)	10 (66.7)	6 (37.5)	
5 + times	7 (23.3)	2 (13.3)	6 (37.5)	
**Other Medical History – Frequency (%)**				
Fussy Eater – yes	28 (90.3)	13 (86.7)	15 (93.8)	0.600
Anxiety – yes	26 (83.9)	12 (80.0)	14 (87.5)	0.654
Depression – yes	5 (16.1)	3 (60.0)	2 (12.5)	0.654
ADHD – yes	19 (61.3)	8 (53.3)	11 (68.8)	0.379
Mood/anxiety or behaviour medication – yes	18 (58.1)	7 (46.7)	11 (68.8)	0.213
Gastrointestinal medication – yes	7 (22.6)	4 (26.7)	3 (18.8)	0.685
Baseline Questionnaires – Mean (SD) / Frequency (%)				
**Primary Outcome**				
6-GSI	5.23 (1.67)	5.40 (1.30)	5.06 (1.99)	0.582
Stool Consistency Hard Normal Loose	Missing = 1 18 (60.0%) 3 (10.0%) 9 (30.0%)	Missing = 1 9 (64.3) 1 (7.1) 4 (28.6)	Missing = 0 8 (50.0) 2 (12.5) 6 (37.5)	0.716
**Baseline Anxiety & Behaviour Scores**				
PRAS-ASD	36.45 (13.0)	30.87 (12.79)	41.69 (11.147)	0.018*
ABC – Irritability	19.84 (9.75)	17.00 (9.62)	22.50 (9.39)	0.118
ABC – Social Withdrawal	10.58 (6.73)	9.20 (5.93)	11.88 (7.35)	0.276
ABC – Stereotypical Behaviour	4.48 (4.27)	4.80 (4.30)	4.19 (4.36)	0.697
ABC- Hyperactivity / Non-compliance	22.45 (10.03)	23.27 (10.04)	21.69 (10.27)	0.669
ABC – Inappropriate Speech	3.90 (2.23)	4.33 (1.99)	3.50 (2.42)	0.306
ABC - Total	61.26 (24.44)	58.60 (24.56)	63.75 (24.86)	0.567

**Key**: ^ calculated using independent t-tests (age and anthropometric data) or chi-squared tests (gender and medical history data); *=statistically significant

*Abbreviation* 6-GSI = 6-item Gastrointestinal Severity Index; ABC = Aberrant Behaviour Checklist; ADHD = Attention Deficient Hyperactivity Disorder; ASD = Autism Spectrum Disorder; BMI = Body Mass Index COM = Combined Treatment Group (Synbiotic + Gut-directed Hypnotherapy); mITT = Modified Intention-to-Treat; PRAS-ASD = Parent-Rated Anxiety Scale – Autism Spectrum Disorder; SD = Standard Deviation; SYN = Synbiotic Treatment Group

### Safety

During the trial, 32 adverse events (AEs) were reported in 22 participants (see Supplementary Table 1). All AEs were followed up to resolution. The majority of AEs were assessed as being unrelated to treatments (20 AEs, 62.5%). The remaining 12 AEs were either possibly (5 AEs) or probably (7 AEs) related to the treatments. The seven probable AEs were transient GI events that resolved early in the trial (SYN group, *n* = 3; COM group, *n* = 4). Of the five possibly related AEs, four were associated with pre-existing GI symptoms that continued (SYN group, *n* = 2; COM group, *n* = 1) or worsened (SYN group, *n* = 1) and the remaining AE was related to mood changes for which suspected hormonal changes could not be verified. Importantly, no serious safety events were associated with this trial.

### Efficacy Results

The two-way ANOVA model showed no significant interactions between treatment group (SYN vs. COM) and time (post-intervention, t = 12 vs. pre-intervention, t = 0) on total 6-GSI scores (*p* = 0.465), ABC subscale scores (ABC-I, *p* = 0.248; ABC-SW, *p* = 0.058; ABC-SB, *p* = 0.450; ABC-HNC, *p* = 0.982; ABC-IS, *p* = 0.405) or anxiety symptoms (*p* = 0.214) (see Table 2), when controlling for sex, baseline anxiety scores and antibiotic exposure. Inclusion of the follow-up timepoint (t = 24) for the primary objective (6-GSI scores) also failed to show a significant association between treatment group and time (*p* = 0.823).

To investigate the effect of any treatment, within group, pairwise comparison was performed for each objective over time. Table 2 provides a summary of the results across two timepoints (pre-intervention [t = 0] to post-intervention [t = 12]). Table 3 provides a summary of the results across all three timepoints (pre-intervention, post-intervention, and follow-up [t = 24]).

**Table 2:** Gastrointestinal, behavioural and anxiety results across two timepoints (pre-intervention [t = 0] & post-intervention [t = 12]), adjusted for sex, pre-intervention anxiety scores & antibiotic exposure

Measurement	2-way ANOVA Treatment*Time	SYN Group					COM Group					Clinically significant threshold^
		Pre-intervention^#^ (t = 0) *n* = 15		Post-intervention^#^ (t = 12) *n* = 15	MD (95% CI)	*p*-value	Pre-intervention^#^ (t = 0) *n* = 16		Post-intervention^#^(t = 12) *n* = 16	MD (95% CI)	*p*-value	
**6GIS**												
Total Score	*F*(1, 27) = 0.550, *p* = 0.465	5.41 (0.48)		2.96 (0.51)	**-2.45** (-3.40, -1.49)	< 0.001*	5.06 (0.46)		2.10 (0.49)	**-2.94** (-3.88, -2.03)	< 0.001*	-2
Constipation	F(1,27) = 0.089, *p* = 0.767	0.97 (0.20)		1.13 (0.22)	0.16 (-0.41, 0.73)	0.570	0.78 (0.20)		0.82 (0.21)	0.04 (-0.51, 0.59)	0.888	Not known
Diarrhoea	F(1,27) = 0.546, *p* = 0.466	0.55 (0.24)		0.00 (0.00)	-0.55 (-1.04, -0.07)	0.027*	0.30 (0.23)		0.00 (0.00)	-0.30 (-0.76, 0.17)	0.205	Not known
Consistency	F(1,27) = 0.041, *p* = 0.842	0.61 (0.16)		0.23 (0.12)	-0.38 (-0.73, -0.03)	0.037*	0.49 (0.17)		0.16 (0.11)	-0.33 (-0.67, 0.01)	0.059	Not known
Smell	F(1,27) = 1.522, *p* = 0.228	1.29 (0.19)		0.72 (0.21)	-0.57 (-1.09, -0.04)	0.035*	1.17 (0.18)		0.14 (0.20)	-1.03 (-1.54, -0.52)	< 0.001*	Not known
Flatulence	F(1,27) = 0.238, *p* = 0.629	0.91 (0.24)		0.52 (0.20)	-0.40(-0.91, 0.13)	0.135	1.14 (0.23)		0.57 (0.20)	-0.57 (-1.07, -0.07)	0.027*	Not known
Pain	F(1,27) = 0.032, *p* = 0.859	1.08 (0.21)		0.36 (0.14)	-0.72 (-1.09, -0.34)	< 0.001*	1.18 (0.21)		0.41 (0.13)	-0.77 (-1.13, -0.41)	< 0.001*	Not known
**ABC**												
Irritability	*F*(1,26) = 1.394, *p* = 0.248	21.17 (1.74)		16.62 (1.87)	-4.55 (-9.24, 0.12)	0.056	18.59 (1.67)		9.99 (1.80)	**-8.60** (-13.11, -4,10)	< 0.001*	-4.65
Social Withdrawal	F(1,26) = 3.930, *p* = 0.058	11.29 (1.69)		9.39 (1.36)	-1.90 (-4.50, 0.69)	0.144	9.92 (1.62)		4.26 (1.31)	**-5.66** (-8.15, -3.16)	< 0.001*	-3.55
Stereotypical Behaviour	F(1,26) = 0.588, *p* = 0.450	5.78 (1.18)		3.80 (0.98)	-1.98 (-3.50, -0.71)	0.004*	3.27 (1.14)		2.00 (0.94)	-1.27 (-2.49, -0.05)	0.042*	-2.10
Hyperactivity/ Non-Compliance	*F*(1,26) = 0.001, *p* = 0.982	26.52 (2.60)		20.10 (2.59)	**-6.42** (-11.00, -1.83)	0.008*	18.64 (2.51)		12.15 (2.50)	**-6.49** (-10.91, -2.07)	0.006*	-5.45
Inappropriate Speech	F(1,26) = 0.715, *p* = 0.405	5.05 (0.50)		4.25 (0.46)	-0.80 (-1.84, 0.25)	0.131	2.83 (0.48)		1.39 (0.44)	**-1.44** (-2.45, -0.43)	0.007*	-1.45
**PRAS-ASD**												
Total	*F*(1,27) = 1.620, *p* = 0.214	32.01 (3.26)		27.24 (3.92)	-4.77 (-2.28, 11.83)	0.176	40.61 (3.15)		29.40 (3.78)	-11.21 (-18.02, -4.41)	0.002*	Not known

**Key** * Statistically significant; # adjusted mean (standard error); ^refer Methods – Measurement Tools; **bold** = clinically significant

*Abbreviation* 6GSI = 6-item Gastrointestinal Severity Index; ABC = Aberrant Behaviour Checklist; CI = Confidence Intervals; COM = Combined Treatment Group (synbiotic + gut-directed hypnotherapy); MD = Mean Difference; PRAS-ASD = Parent-Rated Anxiety Scale – Autism Spectrum Disorder; SYN = Synbiotic Treatment Group; t = x weeks

**Table 3:** Gastrointestinal results across three timepoints (pre-intervention [t = 0], post-intervention [t = 12] & follow-up[t = 24]), adjusted for sex, pre-intervention anxiety scores & antibiotic exposure

Measurement	2-way ANOVA Treatment*Time	SYN Group					COM Group				
		Pre-intervention^#^ (t = 0) *n* = 12	Post-intervention^#^ (t = 12) *n* = 12	Follow-up^#^ (t = 24) *n* = 12	MD (Pre to Follow-Up) (95% CI)	*p*-value	Pre-intervention^#^ (t = 0) *n* = 13	Post-intervention^#^ (t = 12) *n* = 13	Follow-up^#^ (t = 24) *n* = 12	MD (Pre to Follow-Up) (95% CI)	*p*-value
**6GSI**											
Total Score	F(2,40) = 0.196, *p* = 0.823	5.42 (0.58)	2.52 (0.61)	3.14 (0.60)	**-2.28** (-3.92, -0.64)	< 0.001*	5.23 (0.55)	2.06 (0.58)	2.41 (0.57)	**-2.82** (-4.38, -1.26)	< 0.001*
Constipation	F(2,40) = 0.063, *p* = 0.939	1.07 (0.26)	1.33 (0.27)	1.13 (0.27)	0.06 (-0.80, 0.92)	1.000	0.56 (0.25)	0.77 (0.26)	0.73 (0.26)	0.17 (-0.64, 0.99)	1.000
Diarrhoea	F(2,40) = 1.380, *p* = 0.263	0.74 (0.32)	0.00 (0.00)	0.09 (0.07)	-0.65 (-1.34, 0.04)	0.070	0.16 (0.31)	0.00 (0.00)	0.00 (0.00)	-0.17 (-0.83, 0.49)	1.000
Consistency	F(2,40) = 0.257, *p* = 0.775	0.53 (0.21)	0.22 (0.15)	0.36 (0.16)	-0.17 (-0.74, 0.40)	1.000	0.59 (0.20)	0.19 (0.14)	0.21 (0.15)	-0.38 (-0.93, 0.16)	0.249
Smell	F(2,40) = 0.315, *p* = 0.732	1.19 (0.24)	0.51 (0.23)	0.59 (0.24)	-0.59 (-1.29, 0.10)	0.110	1.13 (0.23)	0.14 (0.21)	0.53 (0.23)	-0.61 (-1.27, 0.05)	0.079
Flatulence	F(2,40) = 0.995, *p* = 0.376	0.83 (0.29)	0.26 (0.20)	0.39 (0.20)	-0.44 (-1.10, 0.21)	0.278	1.39 (0.27)	0.45 (0.19)	0.49 (0.19)	-0.90 (-1.52, -0.28)	0.004*
Pain	F(2,40) = 1.202, *p* = 0.311	1.07 (0.24)	0.20 (0.17)	0.58 (0.23)	-0.48 (-1.06, 0.11)	0.139	1.40 (0.23)	0.50 (0.16)	0.46 (0.22)	-0.94	< 0.001*

**Key** * Statistically significant; # adjusted mean (standard error); **bold** = clinically significant (=/> 2 point reduction in total 6GSI; see Methods - Measurement Tools section)

*Abbreviation* 6GSI = 6-item Gastrointestinal Severity Index; CI = Confidence Intervals; COM = Combined Treatment Group (synbiotic + gut-directed hypnotherapy); MD = Mean Difference; SYN = Synbiotic Treatment Group; t = x weeks

#### Primary Outcome – 6-GSI (Gastrointestinal Severity Scores)

Statistically and clinically significant reductions in total 6-GSI scores were observed for both the SYN and COM group from pre-intervention (t = 0) to post-intervention (t = 12) (MD -2.45 [95% CI -3.40, -1.49; *p* < 0.001] and MD -2.94 [95% CI -3.88, -2.03; *p* < 0.001], respectively; Table 2) and from pre-intervention (t = 0) to follow-up (t = 24) (MD -2.28 [95%CI -3.92, -0.64; *p* < 0.001] and MD -2.82 (95% CI -4.38, -1.26; *p* < 0.001], respectively; Table 3), controlling for sex, baseline anxiety scores and antibiotic exposure.

Pairwise comparison of the 6-GSI subdomains (constipation, diarrhea, stool consistency, stool smell, flatulence, and pain) was also conducted. The results revealed that both treatments produced significant improvements in the subdomains of pain (*p* = < 0.001) and stool smell (SYN group, *p* = 0.035; COM group, *p* = < 0.001) post-intervention (Table 2). Additionally, the SYN group showed significant reductions in diarrhea frequency (*p* = 0.027) and improved stool consistency (*p* = 0.037), whereas the COM group experienced a significant reduction in flatulence (*p* = 0.027). The COM group held the post-intervention improvements seen in the gut subdomains of pain (*p* = < 0.001) and flatulence (*p* = 0.004) at follow-up (t = 24), with no significant subdomain findings remaining for the SYN group (Table 3).

#### Other Gut-Based Outcomes

Seventy-three percent of participants in the COM group (*n* = 11) experienced an improvement in stool consistency based on the BSC after 12-weeks, compared to 50% of those in the SYN group (*n* = 6), however, this association was not statistically significant (*p* = 0.212). A non-significant increase in “responders” was also observed in the COM group compared to the SYN group (*n* = 12, 75% vs. *n* = 10, 66.7%, respectively, *p* = 0.704) post-intervention(t = 12). This non-significant trend continued through to follow-up (t = 24) (“responders” COM group *n* = 10, 90.9% vs. SYN group *n* = 7, 63.6%, *p* = 0.311).

#### Secondary Outcomes – Behaviour and Anxiety

As shown in Table 2, the within group analysis found statistically significant associations for the COM group for all outcomes post-intervention, most of which were also clinically significant. Conversely, the SYN group experienced significant reductions in the subscales of ABC-SB and ABC-HNC only, with the latter also reaching clinical significance.

Statistically and clinically significant reductions in ABC-I scores were seen for the COM group after the 12-week intervention (MD -8.60 [95%CI -13.11, -4.10; *p* = < 0.001]), with a trend towards significant association in the SYN group post-intervention (*p* = 0.056). Similar and significant (statistical and clinical) reductions in ABC-HNC scores were seen for both groups after the 12-week intervention (COM group MD -6.49 [95% CI -10.91, -2.07; *p* = 0.006] vs. SYN group MD -6.42 [95% CI -11.00, -1.83; *p* = 0.008]). The COM group also experienced statistically significant reduction in anxiety scores which were not seen in the SYN group post-intervention (MD -11.21, [95% CI -18.02, -4.41; *p* = 0.002] vs. MD -4.77 [95% CI -2.28, 11.83; *p* = 0.176).

#### Per Protocol Analysis of Primary and Secondary Objectives

The findings of the per protocol analysis support the results reported under the mITT analysis - see Supplementary Tables 2 and Supplementary Table 3. As expected, the treatment effect (size of the change/improvement) was greater under the PP analysis compared to the mITT results.

#### Explorative Objective - Stool Microbiome

When comparing changes over time (post-intervention vs. pre-intervention), there was no significant effect on alpha (species richness and Shannon index) in either the SYN or COM group (data not shown). There were, however, several significant changes in microbial composition at family, genus and species level (see Table 4). We did not observe any consistent change to the relative abundance of bacterial taxa in either treatment group at the family or phylum level, although there were significant changes unique to each group at family level. These included an increase in the relative abundance of *Ezakiellaceae* in the SYN group (*p* = 0.037) as well as an increase in relative abundance of *Christensenellaceae* (*p* = 0.40) and *Dialisteraceae* (*p* = 0.17) in the COM group (see Table 4).

**Table 4:** Bacterial taxa showing a significant change (*P* ≤ 0.05) from pre-intervention [t = 0] to post-intervention [t = 12]

Taxa	SYN Group				Taxa	COM Group			
	Pre-intervention^#^ (t = 0)	Post-intervention^#^ (t = 12)	Delta	*p*-value^		Pre-intervention^#^ (t = 0)	Post-intervention^#^ (t = 12)	Delta	*p*-value^
**SPECIES**									
***Bifidobacterium animalis***	-0.68 [-0.79,0.81]	1.6 [-0.59,3.2]	2.28	0.015	*Clostridium_Q sp003024715*	-0.63 [-0.76,2.2]	2.4 [1.9,2.6]	3.03	0.001
*CAG-272 MIC7215 (Oscillospirales)*	-0.63 [-0.72,0.83]	1.6 [-0.58,2.1]	2.23	0.004	***Bifidobacterium animalis***	-0.6 [-0.74,-0.57]	1.9 [-0.52,2.5]	2.50	0.010
*Blautia_A sp000285855*	1.0 [-0.29,1.7]	2.6 [-0.17,3.2]	1.60	0.048	*Faecalibacterium MIC8666*	1.0 [-0.65,3.1]	3.1 [-0.49,3.8]	2.10	0.006
*Alistipes_A indistinctus*	1.5 [-0.64,2.4]	2.4 [-0.59,2.8]	0.90	0.017	*Faecalibacterium prausnitzii_G*	3.8 [3.2,4.9]	4.9 [4.4,5.3]	1.10	0.014
*ER4 sp000765235 (Oscillospiraceae)*	2.6 [0.096,3.5]	3.4 [2.8,4]	0.80	0.006	*Blautia_A massiliensis*	3.1 [2.3,3.9]	4.0 [3,4.4]	0.90	0.006
***GCA-900066135 MIC6659 (Lachnospiraceae)***	2.0 [1.4,2.7]	2.7 [1.8,3.1]	0.70	0.027	*Blautia_A sp900066205*	1.4 [-0.58,2]	2.1 [0.88,2.3]	0.70	0.029
*UBA7160 MIC9207 (Lachnospiraceae)*	1.1 [-0.17,1.4]	1.6 [-0.052,1.8]	0.50	0.001	*Alistipes finegoldii*	1.7 [-0.58,2.9]	2.4 [0.78,3.1]	0.70	0.022
*Oscillibacter MIC9361*	-0.73 [-0.8,-0.64]	-0.59 [-0.8,-0.52]	0.14	0.026	***GCA-900066135 MIC6659 (Lachnospiraceae)***	2.4 [1.5,2.9]	3.0 [2.1,3.2]	0.60	0.050
*Oscillibacter MIC7608*	-0.72 [-0.8,-0.63]	-0.58 [-0.8,0.73]	0.14	0.020	*Intestinibacter bartlettii*	2.7 [1.8,3.5]	3.3 [2.9,3.7]	0.60	0.019
*Oscillospiraceae MIC9346*	-0.72 [-0.79,-0.6]	-0.58 [-0.73,0.46]	0.14	0.023	*Bacteroides stercoris*	3.3 [-0.52,4.3]	3.7 [-0.51,4.3]	0.40	0.018
*Clostridium MIC6904*	-0.72 [-0.79,-0.63]	-0.59 [-0.73,0.48]	0.13	0.039	*Anaerostipes hadrus*	3.8 [3.2,4.5]	4.2 [3.6,4.9]	0.40	0.052
*CAG-170 MIC9129 (Oscillospiraceae)*	-0.67 [-0.77,-0.54]	-0.54 [-0.59,2.1]	0.13	0.046	*Dialister invisus*	4.2 [3.5,4.8]	4.6 [4.1,5.1]	0.40	0.028
*Blautia_A sp900066335*	-0.64 [-0.73,0.99]	-0.58 [-0.73,2.1]	0.06	0.026	*Oscillibacter MIC7430*	-0.67 [-0.76,-0.59]	-0.52 [-0.7,1.1]	0.15	0.019
*Slackia_A piriformis*	-0.68 [-0.79,-0.56]	-0.63 [-0.83,-0.58]	0.05	0.044	*Dorea sp900066555*	-0.66 [-0.76,-0.59]	-0.52 [-0.69,1.3]	0.14	0.011
*Clostridium_M MIC6986*	-0.67 [-0.78,-0.56]	-0.62 [-0.81,1.1]	0.05	0.007	*Prevotella copri*	-0.59 [-0.67,2.1]	-0.52 [-0.66,5.8]	0.07	0.040
*Absiella sp000163515*	-0.73 [-0.8,-0.64]	-0.71 [-0.84,-0.58]	0.02	0.036	*Blautia_A MIC7077*	-0.63 [-0.74,-0.57]	-0.57 [-0.69,1.5]	0.06	0.013
*Dorea sp000509125*	-0.73 [-0.8,-0.64]	-0.71 [-0.84,-0.58]	0.02	0.042	*UBA1191 sp900066305 (Anaerovoracaceae)*	-0.63 [-0.73,-0.57]	-0.62 [-0.67,0.6]	0.01	0.054
*QAMH01 MIC6543 (Coriobacteriales)*	-0.64 [-0.77,-0.54]	-0.63 [-0.8,-0.58]	0.01	0.015	*Monoglobus pectinilyticus*	-0.64 [-0.76,1.8]	-0.66 [-0.81,-0.49]	-0.02	0.025
*COE1 sp001916965 (Lachnospiraceae)*	-0.57 [-0.73,1.6]	-0.58 [-0.73,0.9]	-0.01	0.035	*CAG-269 sp000431335 U (Clostridia)*	-0.63 [-0.73,-0.57]	-0.66 [-0.81,-0.6]	-0.03	0.051
*Adlercreutzia equolifaciens*	-0.59 [-0.76,1.5]	-0.63 [-0.81,0.72]	-0.04	0.045	*Eubacterium callanderi*	-0.6 [-0.76,-0.41]	-0.66 [-0.81,-0.6]	-0.06	0.046
*Phil1 sp001940855 (Christensenellales)*	-0.57 [-0.7,2.4]	-0.63 [-0.8,1.1]	-0.06	0.052	*CAG-353 sp900066885 (Ruminococcaceae)*	-0.6 [-0.73,-0.41]	-0.66 [-0.81,-0.6]	-0.06	0.047
*Bifidobacterium MIC7686*	-0.64 [-0.79,0.72]	-0.71 [-0.84,-0.59]	-0.07	0.055	*Ruminococcus_E sp003526955*	-0.58 [-0.66,2.6]	-0.64 [-0.69,-0.54]	-0.06	0.053
*Roseburia inulinivorans*	3.2 [2.1,3.8]	2.5 [-0.59,3.2]	-0.70	0.047	*CAG-74 MIC8932 (Christensenellales)*	-0.57 [-0.6,2.1]	-0.64 [-0.69,-0.54]	-0.07	0.009
*Clostridium sp000435835*	2.8 [2.3,3.2]	1.9 [0.98,3.3]	-0.90	0.030	*Bacteroides MIC8726*	-0.57 [-0.76,0.97]	-0.64 [-0.81,-0.54]	-0.07	0.034
*Faecalicatena torques*	2.2 [1.4,3.8]	1.3 [-0.8,2.2]	-0.90	0.049	*Clostridium MIC9230*	-0.59 [-0.68,-0.41]	-0.66 [-0.81,-0.6]	-0.07	0.038
*Oscillibacter sp900066435*	1.7 [-0.091,2]	-0.62 [-0.81,1.3]	-2.32	0.011	*CAG-302 sp000431795 (Bacilli)*	-0.59 [-0.68,-0.41]	-0.66 [-0.81,-0.58]	-0.07	0.041
*Bacteroides stercoris*	3.4 [2.3,4.7]	0.68 [-0.59,3.9]	-2.72	0.043	*UBA644 MIC9235 (Oscillospirales)*	-0.59 [-0.68,-0.41]	-0.66 [-0.81,-0.6]	-0.07	0.041
					*Clostridium sp001916075*	-0.47 [-0.66,2.2]	-0.62 [-0.69,-0.49]	-0.15	0.021
					*Bilophila wadsworthia*	2.2 [1.8,2.8]	1.9 [1.5,2.4]	-0.30	0.050
					*Alistipes onderdonkii*	3.1 [1.9,3.9]	2.7 [-0.51,3.2]	-0.40	0.025
					*Coprococcus eutactus_A*	0.51 [-0.64,2.8]	-0.62 [-0.81,-0.49]	-1.13	0.017
					*CAG-110 sp000434635 (Oscillospiraceae)*	0.95 [-0.64,3.1]	-0.57 [-0.66,1.6]	-1.52	0.050
					*Ruminiclostridium_E siraeum*	2.6 [-0.52,3.2]	-0.64 [-0.76,-0.49]	-3.24	0.001
**GENUS**									
*CAG-272 MIC7215 (Oscillospirales)*	-0.96 [-1,0.44]	1.2 [-0.84,1.7]	2.16	0.003	*Prevotella*	-0.86 [-0.98,2.6]	2.1 [-0.85,5.6]	2.96	0.014
*CAG-103 (Oscillospiraceae)*	2.1 [1.5,2.7]	2.9 [2,3.7]	0.80	0.021	*Clostridium_Q*	0.35 [-1,2.2]	2.1 [1.5,2.4]	1.75	0.004
*Alistipes_A*	2.7 [2.2,3.4]	3.2 [2.4,3.5]	0.50	0.036	*Intestinibacter*	2.6 [1.5,3.2]	3.2 [3,3.5]	0.60	0.012
*GCA-900,066,135 (Lachnospiraceae)*	2 [1.3,2.4]	2.4 [1.5,2.8]	0.40	0.027	*Anaerostipes*	3.9 [3.1,4.2]	4.4 [3.6,5]	0.50	0.024
*Dialister*	1.3 [-1,3.4]	1.5 [-0.89,4.5]	0.20	0.049	*Dialister*	3.9 [3.2,4.5]	4.3 [3.8,4.7]	0.40	0.012
*UBA7160 (Lachnospiraceae)*	1.2 [0.63,1.6]	1.4 [0.99,2]	0.20	0.054	*Faecalibacterium*	5.7 [5.1,6]	6 [5.6,6.2]	0.30	0.029
*Oscillospiraceae MIC9346*	-1 [-1.2,-0.91]	-0.86 [-1.1,0.071]	0.14	0.016	*Agathobaculum*	2 [-0.8,2.5]	2.3 [2,2.7]	0.30	0.044
*CAG-274 (Oscillospirales)*	-1.1 [-1.2,-0.98]	-0.98 [-1.2,-0.83]	0.12	0.031	*Adlercreutzia*	1.6 [0.9,2.2]	1.9 [1.3,2.5]	0.30	0.050
*UBA738 (Oscillospiraceae)*	-1 [-1.1,-0.85]	-0.89 [-1.1,-0.82]	0.11	0.053	*CAG-272 MIC8971 (Oscillospirales)*	-0.95 [-1,-0.8]	-0.73 [-0.85,0.98]	0.22	0.043
*Merdibacter*	-1 [-1.2,-0.95]	-0.94 [-1.1,-0.83]	0.06	0.051	*Monoglobus*	-0.89 [-1,1.5]	-0.87 [-1.1,-0.74]	0.02	0.030
*QAMH01 (Coriobacteriales)*	-0.98 [-1.2,-0.78]	-0.94 [-1.1,-0.83]	0.04	0.002	*CAG-353 (Ruminococcaceae)*	-0.91 [-1,-0.69]	-0.92 [-1.1,-0.84]	-0.01	0.053
*COE1 (Lachnospiraceae)*	-0.86 [-1.1,1.3]	-0.86 [-1.1,0.54]	0.00	0.037	*CAG-74 MIC8932 (Christensenellales)*	-0.82 [-0.92,1.8]	-0.86 [-0.98,-0.81]	-0.04	0.010
*Sellimonas*	-1 [-1.2,-0.95]	-1 [-1.2,-0.86]	0.00	0.053	*UBA644 (Oscillospirales)*	-0.88 [-0.98,-0.67]	-0.92 [-1.1,-0.84]	-0.04	0.043
*Holdemania*	-0.96 [-1.1,-0.16]	-1 [-1.2,-0.88]	-0.04	0.042	*Ruminiclostridium_E*	2.3 [-0.81,2.9]	-0.86 [-1.1,-0.74]	-3.16	0.001
*Adlercreutzia*	1.2 [-1,1.7]	0.018 [-1.1,1.2]	-1.18	0.044	*Coprococcus*	3.2 [-0.21,4.8]	-0.73 [-0.85,3.5]	-3.93	0.011
**FAMILY**									
***Ezakiellaceae***	-1.6 [-1.9,-1.4]	-1.5 [-1.7,0.63]	0.10	0.037	*Christensenellaceae*	-1.4 [-1.6,-1.2]	-0.81 [-1.5,-0.012]	0.59	0.040
*CAG-274 (Lachnospirales)*	-1.7 [-1.9,-1.5]	-1.6 [-1.9,-1.3]	0.10	0.041	*Dialisteraceae*	3.2 [2.4,3.9]	3.7 [3,4.1]	0.50	0.017
*Acutalibacteraceae*	5.6 [4.8,5.9]	5.3 [4.8,5.5]	-0.30	0.054	*Ruminococcaceae*	5.6 [5,5.9]	5.8 [5.4,6.1]	0.20	0.036
					***Ezakiellaceae***	-1.3 [-1.5,1.2]	-1.4 [-1.7,-1.4]	-0.10	0.026
					*CAG-611 (Bacilli)*	-1.3 [-1.5,2.6]	-1.4 [-1.5,-0.8]	-0.10	0.035
					*UBA644 (Oscillospirales)*	-1.4 [-1.6,-1.2]	-1.5 [-1.7,-1.4]	-0.10	0.043

*Abbreviations* COM = Combined Treatment Group (synbiotic + gut-directed hypnotherapy); SYN = Synbiotic Treatment Group; t = x weeks

Key # centroid log ratio data, medium [interquartile range]; Delta = magnitude of changes from pre- to post-intervention (positive value shows an increase while negative value shows a decrease)

^ p-values are based on LMER (linear mixed effect regression) and were not corrected for the multiplicity of testing

At the genus level, the relative abundance of *Dialister* was significantly increased in both the SYN and COM groups (*p* = 0.049 and *p* = 0.012, respectively). Moreover, there were significant changes to the relative abundance in genera unique to each group such as an increase in *Oscillospirales (CAG-272 MIC7215)* (*p* = 0.003) and *Oscillospiraceae (CAG-103)* (*p* = 0.021) in the SYN group as well as an increase in *Prevotella* (*p* = 0.014), *Clostridium Q* (*p* = 0.004) and *Faecalibacterium* (*p* = 0.029) in the COM group.

At species level, among the most prominent changes observed for both treatment groups (SYN and COM) were a consistent increase in the relative abundance of *Bifidobacterium animalis* (*p* = 0.015 and *p* = 0.010, respectively) and an increase in the relative abundance of a species belonging to the family *Lachnospiraceae* (GCA-900066135 MIC6659) (*p* = 0.027 and *p* = 0.050, respectively) In addition, there were significant increases in relative abundance of several, but different, species belonging to *Blautia*,* Oscillibacter* and *Clostridium* in both treatment groups. There were also significant changes unique to each group such as an increase in the relative abundance of *Oscillospirales (CAG-272 MIC7215)* (*p* = 0.004) and *Oscillospiraceae (ER4 sp000765235)(p* *= 0.006)* in the SYN group as well as an increase in the relative abundance of *Faecalibacterium MIC8666* (*p* = 0.006) and *Faecalibacterium prausnitzii G* (*p* = 0.014) in the COM group (see Table 4).

## Discussion

A combined approach, administering an oral synbiotic in conjunction with a home-based gut-directed hypnotherapy (GDH) program (COM group), was not superior to synbiotics alone (SYN group) at reducing GI symptom severity, behaviour or anxiety scores in autistic children with comorbid DGBI across a 12-week intervention. While there was no difference between the groups over time, both treatments were independently associated with statistically and clinically significant reductions in GI symptom severity across the 12-week intervention. Interestingly, the addition of GDH (COM group) significantly improved pain and flatulence outcomes at follow-up, indicating a combined approach may improve the longevity of beneficial outcomes for specific GI symptoms. Moreover, the COM group experienced statistically significant (and many clinically significant) reductions in all behavioural (ABC) subdomains, as well as significant reductions in anxiety scores, that were not evident in the SYN group.

These findings are consistent with those reported in previous trials on probiotic interventions in autistic children, however, different probiotics combinations and trial methodologies makes comparing results difficult. West et al. found that 21 days of Delpro^®^ (1 × 10^8 CFUs of L. *acidophilus*,* L. casei*,* L.delbrueckii*,* B. longum*,* B. bifidum* + immunomodulator Del-Immunie V^®^) decreased the severity of GI symptoms (constipation and diarrhea) and total behavioural scores in participants (3–16 years, *n* = 33) during their open-label study (West et al., 2013). Shaaban et al., found that a 3-month intervention with 5 g per day of *L. acidophilus*,* L.rhamnosus B. longum* (per gram contains 100 × 10^6 CFUs) significantly reduced total gut severity scores (*p* < 0.001) and the subdomains of abdominal pain (*p* < 0.002), constipation (*p* < 0.01), flatulence (*p* < 0.037), and stool consistency (*p* < 0.023) in autistic children (aged 5–9 years, *n* = 30) (Shaaban et al., 2018). Reduced pain outcomes were also reported by Sanctuary et al., (Sanctuary et al., 2019) after 10 weeks of *B. infantis (UCD272*,* 20 billon CFUs/day)* with or without bovine colostrum (BC) (Imucon, 0.15 g/lb body weight/day in autistic children (2–11 years, *n* = 8). BC acts a prebiotic in the gut and administration of this product alone resulted in significant improvements in total behavioural scores and reduced frequency of diarrhea that were not seen in the BC + probiotic group. Another study investigating the benefits of a prebiotic on gut function in autistic children (aged 4–9 years, *n* = 13) was conducted by Inoue et al., (Inoue et al., 2019). In their prospective, open-label study, the researchers administered 6 g/day of PHGG and found children experienced significant improvements in defecation and irritability scores (*p* < 0.01).

Our findings are also supported by those of two controlled studies. Arnold and colleagues (Arnold et al., 2019) conducted a placebo-controlled, cross-over, pilot study that found significant improvements in parent-targeted GI symptoms in autistic children (3–12 years, *n* = 13) treated with Visbiome^®^ (1/2 packet bidaily – 90 × 10^10 CFUs/packet of *L. acidophilus*,* L. plantarum*,* L.paracasei*,* L. delbrueckii subsp. Bulgaricus*,* S. thermpphilus*,* B. longum*,* B. breve*,* B. infantis*) compared to those who received the placebo (*p* = 0.02, *d* = 0.79). Most recently, an RCT by Santocchi and colleagues (Santocchi et al., 2020) (26) found that Visbiome^®^ (2 packets/day in the first month then 1 pacekt/day for 5 months) significantly improved GI symptoms only in autistic children with GI issues, while those with no GI complaints experienced improvements in behaviour that were not observed in the GI subgroup. This study highlights the importance of addressing DGBI in this cohort as a means of supporting behavioural and functional improvements.

To the best of our knowledge this is the first study to investigate a combined gut-brain approach in autistic children with DGBI and one of the few studies to include a follow-up period. This novel approach has produced some promising preliminary results. Although not statistically significant, the COM group produced more treatment responders than the SYN group which could be clinically significant. Importantly, however, the COM group was the only group to show statistically significant reduction in key areas of irritability and anxiety scores. This could be of clinical significance as research shows that both behaviour and anxiety are positively correlated to the severity of DGBI in children (Karagözlü et al., 2021; Mazurek et al., 2013). Moreover, this relationship is likely to be bi-directional (Koloski et al., 2012), meaning improving either GI symptoms or anxiety/irritability could improve the other symptom. Additionally, the COM group maintained statistically and clinically significant improvements in the subdomains of gut pain and flatulence at follow-up (t = 24 weeks), which were not observed in the SYN group. The ability of GDH to promote longer-term benefits has been previously reported by Vlieger and colleagues (Vlieger et al., 2007, 2012). In their initial research, Vlieger et al. conducted an RCT comparing GDH with standard care (SC) in neurotypical children with functional abdominal pain (FAP) and/or irritable bowel syndrome (aged 8–18 years). Findings revealed that GDH was superior to SC with significantly greater reductions in pain scores (*p* = 0.001) after a 3-month intervention (Vlieger et al., 2007). At follow-up (1 year and 5 years) GDH remained superior to SC with significantly more children remaining in clinical remission after treatment (1 year 85% vs. 25% (*p* = 0.001); 5 years 65% vs. 20% (*p* = 0.005), respectively) (Vlieger et al., 2007, 2012). The potential for adjunct GDH to provide longer-term relief from pain after a 12-week treatment period is a valuable outcome for the child and family over ongoing daily treatment from supplementation.

With regard to microbiome profiling, the most noticeable patterns were consistent increases in the relative abundance of *Bifidobacterium* and *Dialister* in both the SYN and COM group while there was a unique increase in the relative abundance of *Faecalibacterium* in the COM group. The increase in relative abundance of *Dialister* is interesting given that beneficial effects have been linked to increases in *Dialister* in previous studies. For example, in a clinical study by Martinez and colleagues (Martínez et al., 2013), healthy humans consumed whole-grain barley, brown rice, or an equal mixture of the two, and characterised their impact on stool microbial ecology and blood markers of inflammation. They found that subjects with greater reduction in IL-6 levels harboured significantly higher proportions of *Dialister* in response to the whole-grain barley intervention. Also, patients with Crohn’s disease display a decrease in Dialister (Marie et al., 2011). Based on these findings, it could be speculated that the increase in *Dialister* was involved in the beneficial GI effects observed in this study. With respect to the consistent increase in *Bifidobacteria*, this outcome could be of clinical significance, as research indicates that autistic children have lower levels of this commensal bacterial species compared to non-autistic controls (Coretti et al., 2018; Wang et al., 2011). Indeed, the health benefits of the genus *Bifidobacterium* are well established and have been discussed in several reviews (He et al., 2023). However, the results of this study differed from Inoue and colleagues open label study investigating a PHGG supplement in autistic children with constipation (Inoue et al., 2019). In this study, 8 weeks of 6 g of PHGG supplementation per day significantly shifted multiple bacterial genera and species, but this did not include *Bifidobacterium*. This variation could be multifactorial, relating to synergistic effect of the prebiotic (PHGG) and probiotic supplement used in this study; differences due to the constipation specific microbial profile present in the cohort studied by Inuoe and colleagues; or due to the inherent individual variation seen in the gastrointestinal microbiome. However, in agreement with our results for the COM group, Inoue and colleagues found statistically significant reductions in irritability scores post PHGG supplementation.

Interestingly, we found several inconsistent changes that were unique to each group such as a significant increase in the relative abundance of *Faecalibacterium* in the COM group only. *Faecalibacterium* has been suggested by many investigations to help maintain gut physiology and host wellbeing (Lopez-Siles et al., 2017) and have been shown to be statistically lower in autistic children compared to non-autistic controls (Agarwala et al., 2021). Moreover, low abundance of *Faecalibacterium prausnitzii* have been shown in several health conditions such as IBD, IBS, diabetes and obesity, as well in mental health disorders such as anxiety and depression (Leylabadlo et al., 2020; Nandwana et al., 2022; Warren et al., 2024). Indeed, high occurrence of DGBI (Madra et al., 2020) and anxiety are reported in ASD (Avni et al., 2018) and are positively correlated (Mazurek et al., 2013). Pre-clinical research has demonstrated that stress and the resulting circulating cortisol can impact gut barrier integrity and modulate the gut bacteria (Tetel et al., 2018). As *Faecalibacterium* is associated with anxiety, the ability for GDH to reduce anxiety and hence reduce circulating cortisol, may help explain the difference seen between groups. The potential for brain-based interventions to modulate the microbiome has been reported previously. For example, a 10-week group GDH program in IBS found small but significant changes in several taxa before and after treatment when not controlling for multiple comparison (Peter et al., 2018). Furthermore, a recent study found evidence of a brain-to-gut effect after implementing a mindfulness-based cognitive therapy in patients with anxiety (Wang et al., 2022). Results found the intervention significantly increased microbial diversity and shifted the microbiome profile closer to that observed in healthy controls, while attenuating anxiety traits. However, the reported changes in bacterial taxa contrasted with our findings, with *Faecalibacterium* levels decreasing post intervention. Hence, what requires consideration is that the observed effects in the COM group are most likely an interaction effect of the synbiotic and GDH which may explain the observed differences in microbiota changes.

The study found that treatments were safe and well tolerated, with the majority of related AEs being mild and transient in nature. These events were likely related to the participant’s gastrointestinal tract adjusting to the introduction of the synbiotic. Transient discomfort due to probiotic interventions has been previously reported in the literature (Santocchi et al., 2020).

This study has some limitations that should be highlighted. First, there was no control group for either treatment group. This leaves the results vulnerable to placebo effects. However, as both treatment arms included the synbiotic supplement, results can assess the additional benefit that a combined approach may bring which was the hypothesis being tested. These results need to be validated in randomised controlled trials before they have clinical use. A second limitation of the study was that randomisation failed to evenly distribute the participants on the basis of sex and baseline anxiety scores. It is well known that males are highly represented among autistic individuals. Therefore, the study sample is representative of the ASD population (i.e. results are generalisable), however, evidence suggests that biological sex differences can impact microbial composition from birth (Valeri & Endres, 2021). Hence, sex may impact the effectiveness of a gut modulating therapy such as a probiotic/prebiotic supplement. If randomisation was successful for sex, these differences would have been controlled for between groups. However, as the proportion of males to females was not even between groups (77.8% vs. 44.4% males in SYN and COM groups respectively, *p* = 0.004), sex was added as a covariant to the analysis in an attempt to adjust for this potential confounder. Baseline anxiety scores were also added to the analysis as a covariant given the statistically significant differences between groups. This was deemed necessary, as anxiety is positively associated with increased GI issues (Mazurek et al., 2013), and those with higher anxiety may be more likely to response to the anxiety/stress reduction elements of the GDH program. The third limitation is the potential for measurement bias with the use of parent-reported tools. While it is common in ASD research to use parent-reported tools, subjective tools (administered by a qualified clinician), could help eliminate the potential for measurement bias. However, this process was not feasible for financial reasons. In addition, clinician led assessments would have placed an additional burden on the parent/guardians and increased participant stress. To help avoid measurement issues, every parent/guardian was given the same, clear instructions for completing the questionnaires/surveys. This field would benefit from standardising the large number of parent-reported tools to facilitate comparison of outcomes between studies. A fourth limitation of the research is the lack of dietary data. We aimed to mitigate the potential confounding influence of diet on the study results by requesting parents/guardians did not make significant changes their child’s diet during their enrolment. This was followed-up during the monitoring process (every 2 weeks) and at study conclusion no parents/guardians had reported any dietary change. Also, as many of autistic children have rigid, repetitive eating patterns (Yap et al., 2021), the risk of significant dietary change was considered low. However, without dietary tracking, dietary change during enrolment cannot be known or controlled for and, therefore, is a limitation of the research that needs to be considered. Finally, as would be expected, a number of participants did not complete the study and we were unable to meet the recruitment target of 20 participants per group. However, it should be remembered that the recruitment target was increased by 25% (16 to 20) in each group to allow for the potential effects of dropouts on the results. Accordingly, the number of participants to complete the 12-week assessment was sufficient in the COM group with 16 participants at 12-weeks, while the SYN group had 15 participants. By the 24-week assessment, however, both groups had dropped below the 16 subjects, which may impact marginally on the findings.

In conclusion, we demonstrated that synbiotic supplementation, with or without GDH, could prove a promising adjunct therapy for autistic children with comorbid DGBI. The results from this study found both treatments to be safe and effective at reducing GI discomfort in autistic children with beneficial microbial shifts evident post-intervention. Moreover, the potential for GDH to produce additional benefits in the key outcomes of irritability and anxiety and to improve the longevity of specific gut symptoms is a novel outcome found in this study with potential clinical relevance. These results should be replicated in randomised controlled trials.

## Electronic supplementary material

Below is the link to the electronic supplementary material.

Supplementary Material 1

Supplementary Material 2

Supplementary Material 3

## Abbreviations

6GSI: 6-item Gastrointestinal Severity Index
ABC: Aberrant Behaviour Checklist
CI: Confidence Intervals
COM: Combined Treatment Group (synbiotic + gut-directed hypnotherapy)
MD: Mean Difference
PRAS-ASD: Parent-Rated Anxiety Scale – Autism Spectrum Disorder
SYN: Synbiotic Treatment Group
t: x weeks
