# Correction: Gastrointestinal, Behaviour and Anxiety Outcomes in Autistic Children Following an Open Label, Randomised Pilot Study of Synbiotics vs Synbiotics and Gut-Directed Hypnotherapy

**DOI:** 10.1007/s10803-024-06632-8

**Published:** 2024-11-08

**Authors:** Leanne K. Mitchell, Helen S. Heussler, Christopher J. Burgess, Ateequr Rehman, Robert E. Steinert, Peter S.W. Davies

**Affiliations:** 1 Child Health Research Centre, Faculty of Medicine, The University of Queensland, South Brisbane, QLD Australia; 2 Child Development Program, Children’s Health Queensland, Brisbane, QLD Australia; 3 Centre for Clinical Trials in Rare Neuro Developmental Disorders, Children’s Health Queensland, Brisbane, QLD Australia; 4 Department of Gastroenterology, Hepatology and Liver Transplant, Queensland Children’s Hospital, Brisbane, QLD Australia; 5 DSM-Firmenich, Health, Nutrition & Care (HNC), Kaiseraugst, Switzerland; 6 Department of Surgery, Division of Visceral and Transplantation Surgery, University Hospital Zurich, Zurich, Switzerland

**Correction: Journal of Autism and Developmental Disorders**

10.1007/s10803-024-06588-9

In this article the probiotic strain details have been incorrectly published with a comma but should be without comma.

In the “Introduction” section last paragraph the text “Humiome®L. rhamnosus GG, Humiome®L.” should have been appeared as “Humiome® L. rhamnosus GG Add - (ATCC 53103), Humiome® L.”

In the “Study Intervention” section second paragraph “Humiome®L. rhamnosus GG, Humiome®L.” should have been appeared as “Humiome® L. rhamnosus GG Add - (ATCC 53103), Humiome® L.”

In the “Introduction” section second paragraph the text “LGG” should have been appeared as “L. rhamnosus GG”.

The original article has been corrected.

