# Supplementary material for: Gastrointestinal, Behaviour and Anxiety Outcomes in Autistic Children Following an Open Label, Randomised Pilot Study of Synbiotics vs Synbiotics and Gut-Directed Hypnotherapy
Source: J Autism Dev Disord. 2024 Oct 17;56(3):1027–45. doi: 10.1007/s10803-024-06588-9 (PMC12935829; doi:10.1007/s10803-024-06588-9)
Supplement: Supplementary file 1 — Supplementary Material 1 [file 10803_2024_6588_MOESM1_ESM.docx]

**Supplementary Table 1: Adverse Events Reported During Trial**

|  | **Participant Code** | **Allocation** | **Body System** | **Adverse Event** | **Duration** | **Severity**  Mild  Moderate  Severe | **Seriousness**  Serious  Non-serious | **Relatedness**  Possible  Probable  Definite  Unrelated | **Action**  None  Dosage change  Interrupted  Withdrawn | **Outcome**  Resolved  Ongoing -stable  Unresolved  Withdrawn |
| --- | --- | --- | --- | --- | --- | --- | --- | --- | --- | --- |
| 1 | ASD-MGB-001 | COM | GI – New | Flatulence | ~14 days | Mild | Non-serious | Probable | None | Resolved |
| 2 | ASD-MGB-002 | SYN | GI - Unrelated | Infection* | 4 days | Mild | Non-serious | Unrelated | None | Resolved |
| 3 | ASD-MGB-007 | COM | GI - Unrelated | Infection* | 7 days | Mild | Non-serious | Unrelated | None | Resolved |
| 4 | ASD-MGB-007 | COM | Immune | Hayfever | Across enrolment | Mild | Non-serious | Unrelated | None | Ongoing - stable |
| 5 | ASD-MGB-007 | COM | Immune | Cold/flu | 3 days | Mild | Non-serious | Unrelated | None | Resolved |
| 6 | ASD-MGB-007 | COM | Respiratory | Asthma | 3 days | Mild | Non-serious | Unrelated | None | Resolved |
| 7 | ASD-MGB-008 | COM | Dermatological | Molluscum | 8 weeks | Mild | Non-serious | Unrelated | None | Resolved |
| 8 | ASD-MGB-008 | COM | Brain/behaviour | Irritation^ | 10 days | Mild | Non-serious | Unrelated | None | Resolved |
| 9 | ASD-MGB-009 | SYN | GI – Pre-existing | Pain, stool consistency, incontinence | 6 weeks | Mild | Non-serious | Possible | Withdrawn | Withdrawn - stabilised |
| 10 | ASD-MGB-010 | SYN | GI - New | Diarrhoea | 7 days | Mild | Non-serious | Probable | None | Resolved |
| 11 | ASD-MGB-013 | COM | GI – Pre-existing | Constipation+ pain |  | Mild | Non-serious | Possible | None | Ongoing - stable |
| 12 | ASD-MGB-013 | COM | GI - Unrelated | Diarrhoea + vomit | 1 day | Mild | Non-serious | Unrelated | None | Resolved |
| 13 | ASD-MGB-014 | COM | GI - Unrelated | Diarrhoea, vomit + fever* | 10 days | Mild | Non-serious | Unrelated | None | Resolved |
| 14 | ASD-MGB-014 | COM | Brain/behaviour | Irritation# | 6 weeks | Mild | Non-serious | Unrelated | None | Resolved |
| 15 | ASD-MGB-015 | SYN | GI - New | Increased frequency & urgency | 7 days | Mild | Non-serious | Probable | None | Resolved |
| 16 | ASD-MGB-015 | SYN | Immune | Cold | 6 days | Mild | Non-serious | Unrelated | None | Resolved |
| 17 | ASD-MGB-015 | SYN | Dental | Tooth decay | N/A | Mild | Non-serious | Unrelated | None | Resolved |

**Supplementary Table 1: Adverse Events Reported During Trial - continued**

|  | **Participant Code** | **Allocation** | **Body System** | **Adverse Event** | **Duration** | **Severity**  Mild  Moderate  Severe | **Seriousness**  Serious  Non-serious | **Relatedness**  Possible  Probable  Definite  Unrelated | **Action**  None  Dosage change  Interrupted  Withdrawn | **Outcome**  Resolved  Ongoing -stable  Unresolved  Withdrawn |
| --- | --- | --- | --- | --- | --- | --- | --- | --- | --- | --- |
| 18 | ASD-MGB-016 | SYN | Brain/behaviour | Anxiety & Irritation | Across enrolment | Mild | Non-serious | Possible | None | Resolved |
| 19 | ASD-MGB-018 | COM | Immune | Sinus infection | 3 days | Mild | Non-serious | Unrelated | None | Resolved |
| 20 | ASD-MGB-019 | SYN | GI – Pre-existing | Constipation | 6 weeks | Mild | Non-serious | Possible | Withdrawn | Withdrawn - stabilised |
| 21 | ASD-MGB-025 | COM | GI - Unrelated | Diarrhoea + vomit* | 2 days | Mild | Non-serious | Unrelated | None | Resolved |
| 22 | ASD-MGB-027 | COM | GI – Pre-existing | Pain related to anxiety | 3 days | Mild | Non-serious | Possible | None | Resolved |
| 23 | ASD-MGB-029 | SYN | GI – Pre-existing | Reflux/diarrhoea dairy exposure | 2 days | Mild | Non-serious | Unrelated | None | Resolved |
| 24 | ASD-MGB-029 | SYN | GI - Unrelated | Infection* | 6 days | Mild | Non-serious | Unrelated | None | Resolved |
| 24 | ASD-MGB-030 | SYN | GI – Pre-existing | Loose stools | 6 weeks | Mild | Non-serious | Possible | None | Resolved |
| 26 | ASD-MGB-030 | SYN | Immune | Cold/Flu | 8 days | Mild | Non-serious | Unrelated | None | Resolved |
| 27 | ASD-MGB-031 | COM | GI – New | Pain before stool | 3 weeks | Mild | Non-serious | Probable | None | Resolved |
| 28 | ASD-MGB-032 | SYN | GI – Pre-existing | Pain + bloating gluten exposure | 2 weeks | Mild | Non-serious | Unrelated | None | Resolved |
| 29 | ASD-MGB-033 | COM | GI – New | Flatulence | Throughout | Mild | Non-serious | Probable | None | Ongoing - stable |
| 30 | ASD-MGB-034 | COM | GI – New | Diarrhoea | 2 days | Mild | Non-serious | Probable | None | Resolved |
| 31 | ASD-MGB-035 | SYN | Brain/behaviour | Behaviour change linked to changing medication | 3 weeks | Mild | Non-serious | Unrelated | None | Resolved |
| 32 | ASD-MGB-039 | COM | GI – New | Frequency + consistency | 3 weeks | Mild | Non-serious | Probable | None | Resolved  NB later withdrew for non-compliance |

***Key:*** ** = others in household with the same symptoms; ^=change in routine due to COVID lockdown; #=school stress, sleep issues, medication change*

***Abbreviations****: COM = Combined Treatment Group (Synbiotic + Gut-Directed Hypnotherapy); GI = Gastrointestinal; SYN = Synbiotic Treatment Group*
