# Supplementary material for: Gastrointestinal, Behaviour and Anxiety Outcomes in Autistic Children Following an Open Label, Randomised Pilot Study of Synbiotics vs Synbiotics and Gut-Directed Hypnotherapy
Source: J Autism Dev Disord. 2024 Oct 17;56(3):1027–45. doi: 10.1007/s10803-024-06588-9 (PMC12935829; doi:10.1007/s10803-024-06588-9)
Supplement: Supplementary file 2 — Supplementary Material 2 [file 10803_2024_6588_MOESM2_ESM.docx]

| **Measurement** | **2-way ANOVA**  **Treatment*Time** | **SYN Group** | | | | | **COM Group** | | | | | **Clinically significant threshold^** |
| --- | --- | --- | --- | --- | --- | --- | --- | --- | --- | --- | --- | --- |
|  |  | Pre-intervention^#^ (t=0) n=15 | | Post-intervention^#^ (t=12) n=15 | MD (95% CI) | *p*-value | Pre-intervention^#^ (t=0) n=16 | | Post-intervention^#^(t=12) n=16 | MD (95% CI) | *p*-value |  |
| **6GIS** |  |  |  |  |  |  |  |  |  |  |  |  |
| Total Score | F(1,17) = 0.147, *p*=0.706 | 5.66 (0.59) | | 2.64 (0.71) | **-3.02**  (-4.19, -1.84) | <0.001* | 5.25 (0.59) | | 1.90 (0.71) | **-3.35**  (-4.52, -2.18) | <0.001* | -2 |
| Constipation | F(1,17) = 2.167,  *p*=0.159 | 0.79 (0.25) | | 1.32 (0.29) | 0.53  -0.19, 1.26) | 0.139 | 0.94 (0.25) | | 0.68 (0.29) | -0.26  (-0.98, 0.46) | 0.460 | Not known |
| Diarrhoea | F(1,17) = 1.176, *p*=0.293 | 0.89 (0.35) | | 0.00 (0.00) | -0.89  (-1.62, -0.15) | 0.021* | 0.30 (0.35) | | 0.00 (0.00) | -0.30  (-1.03, 0.44) | 0.409 | Not known |
| Consistency | F(1,17) = 0.271, *p*=0.609 | 0.66 (0.22) | | 0.34 (0.16) | -0.32  (-0.79, 0.15) | 0.169 | 0.61 (0.22) | | 0.11 (0.16) | -0.50  (-0.97, -0.03) | **0.038** | Not known |
| Smell | F(1, 17) = 1.207, *p*=0.287 | 1.11 (0.21) | | 0.47 (0.24) | -0.64  (-1.30, 0.02) | 0.055 | 1.26 (0.21) | | 0.08 (0.24) | -1.18  (-1.83, -0.52) | 0.001* | Not known |
| Flatulence | F(1,17) = 0.279, *p*=0.606 | 1.01 (0.32) | | 0.32 (0.24) | -0.69  (-1.44, -0.06) | 0.069 | 1.17 (0.32) | | 0.77 (0.24) | -0.40  (-1.15, 0.35) | 0.279 | Not known |
| Pain | F(1,17) = 0.854, *p*=0.368 | 1.20 (0.24) | | 0.19 (0.16) | -1.01  (-1.43, -0.59) | <0.001* | 0.98 (0.24) | | 0.26 (0.16) | -0.72  (-1.14, -0.30) | 0.002* | Not known |
| **ABC** |  |  | |  |  |  |  | |  |  |  |  |
| Irritability | F(1,17) = 2.838, *p*=0.110 | 19.02 (1.89) | | 14.95 (1.82) | -4.07  (-9.63, 1.49) | 0.141 | 17.71 (1.89) | | 6.70 (1.82) | **-11.01**  (-16.58, -5.47) | <0.001* | -4.65 |
| Social Withdrawal | F(1,17) = 1.324, *p*=0.266 | 10.58 (2.21) | | 7.84 (1.53) | -2.74  (-0.43, 5.90) | 0,085 | 8.60 (2.21) | | 3.17 (1.53) | **-5.43**  (-8.61, 2.28) | 0.002* | -3.55 |
| Stereotypical Behaviour | F(1,17) = 0.10, *p*=0.923 | 5.77 (1.56) | | 4.24 (1.28) | -1.53  (-3.23, 0.17) | 0.075 | 3.15 (1.56) | | 1.49 (1.28) | -1.66  (-3.36, 0.49) | 0.056 | -2.10 |
| Hyperactivity/  Non-Compliance | F(1,17) = 0.065, *p*=0.801 | 27.89 (3.11) | | 21.26 (3.19 | **-6.63**  (-12,48, -0.77) | 0.029* | 16.20 (3.11) | | 8.47 (3.19) | **-7.73**  (-13.60, -1.89) | 0.013* | -5.45 |
| Inappropriate Speech | F(1,17) = 1.750, *p*=0.203 | 5.11 (0.66) | | 4.40 (0.61) | -0.71  (-2.04, 0.63) | 0.278 | 2.98 (0.66) | | 0.96 (0.61) | **-2.02**  (-3.35, -0.69) | 0.005* | -1.45 |
| **PRAS-ASD**  Tota | F(1,18) = 1.471, *p*=0.241 | 29.70 (4.04) | | 25.07 (4.26) | -4.63  (14.37, 5.12) | 0.332 | 38.06 (4.04) | | 24.75 (4.26) | -13.28  (-23.03, -3.53) | 0.010* | Not known |
|  |  |  | |  |  |  |  | |  |  |  |  |

Supplementary Table 2: Per Protocol Analysis of Gastrointestinal, behavioural and anxiety results across two timepoints (pre-intervention [t=0] & post-intervention [t=12]), adjusted for sex, pre-intervention anxiety scores & antibiotic exposure

***Key:*** ** Statistically significant; # adjusted mean (standard error); ^refer Methods – Measurement Tools;* ***bold*** *= clinically significant*

***Abbreviations:*** *6GSI = 6-item Gastrointestinal Severity Index; ABC = Aberrant Behaviour Checklist; CI = Confidence Intervals; COM = Combined Treatment Group (synbiotic + gut-directed hypnotherapy); MD = Mean Difference; PRAS-ASD = Parent-Rated Anxiety Scale – Autism Spectrum Disorder; SYN = Synbiotic Treatment Group; t = x weeks*
