# Supplementary material for: Gastrointestinal, Behaviour and Anxiety Outcomes in Autistic Children Following an Open Label, Randomised Pilot Study of Synbiotics vs Synbiotics and Gut-Directed Hypnotherapy
Source: J Autism Dev Disord. 2024 Oct 17;56(3):1027–45. doi: 10.1007/s10803-024-06588-9 (PMC12935829; doi:10.1007/s10803-024-06588-9)
Supplement: Supplementary file 3 — Supplementary Material 3 [file 10803_2024_6588_MOESM3_ESM.docx]

Supplementary Table 3: Per Protocol Analysis of Gastrointestinal results across three timepoints (pre-intervention [t=0], post-intervention [t=12] & follow-up[t=24]), adjusted for sex, pre-intervention anxiety scores & antibiotic exposure

| **Measurement** | **2-way ANOVA**  **Treatment*Time** | **SYN Group** | | | | | **COM Group** | | | | |
| --- | --- | --- | --- | --- | --- | --- | --- | --- | --- | --- | --- |
|  |  | Pre-intervention^#^ (t=0) n=12 | Post-intervention^#^ (t=12) n=12 | Follow-up^#^ (t=24) n= 12 | MD (Pre to Follow-Up)  (95% CI) | *p*-value | Pre-intervention^#^ (t=0) n=13 | Post-intervention^#^ (t=12) n=13 | Follow-up^#^ (t=24) n= 12 | MD (Pre to Follow-Up)  (95% CI) | *p*-value |
| **6GSI** |  |  |  |  |  |  |  |  |  |  |  |
| Total Score | F(1,13) = 0.020, *p*=0.890 | 5.37 (0.67) | 2.32 (0.82) | 2.69 (0.76) | **-2.68**  (-4.62, -0.73) | 0.007* | 6.08 (0.67) | 2.12 (0.82) | 2.64 (0.76) | **-3.43**  (-5.38, -1.49) | <0.001* |
| Constipation | F(1,13) = 0.941, *p*=0.350 | 0.83 (0.29) | 1.48 (0.45) | 1.07 (0.37) | 0.25  (-0.87, 1.36) | 1.000 | 0.73 (0.29) | 0.74 (0.35) | 0.71 (0.37) | -0.02  (-1.14, 1.09) | 1.000 |
| Diarrhoea | F(1,13) = 1.347 = *p*=0.267 | 1.03 (0.44) | 0.00 (0.00) | 0.13 (0.10) | -0.90  (-1.89, 0.09) | 0.080 | 0.20 (0.44) | 0.00 (0.00) | 0.00 (0.10) | -0.21  (-1.20, 0.78) | 1.000 |
| Consistency | F(1,13) = 0.008, *p*=0.928 | 0.52 (0.26) | 0.28 (0.19) | 0.28 (0.19) | -0.24  (-0.96, 0.49) | 1.000 | 0.82 (0.26) | 0.17 (0.19) | 0.17 (0.19) | -0.65  (-1.38, 0.07) | 0.085 |
| Smell | F(1,13) = 0.458, *p*=0.510 | 0.97 (0.25) | 0.26 (0.24) | 0.38 (0.27) | -0.59  (-1.32, 0.12) | 0.122 | 1.36 (0.25) | 0.18 (0.24) | 0.63 (0.27) | -0.74  (-1.46, -0.02) | 0.044* |
| Flatulence | F(1,13) = 1.283, *p*=0.278 | 0.97 (0.34) | 0.23 (0.25) | 0.42 (0.23) | -0.54  (-1.46, 0.37) | 0.382 | 1.59 (0.34) | 0.66 (0.25) | 0.58 (0.23) | -1.01  (-1.93, -0.10) | 0.028* |
| Pain | F(1,13) = 0.848, *p*=0.374 | 1.06 (0.23) | 0.07 (0.18) | 0.42 (0.27) | -0.65  (-1.39, 0.10) | 0.098 | 1.38 (0.23) | 0.38 (0.18) | 0.58 (0.27) | -0.80  (-1.54, -0.06) | 0.034* |

***Key:*** ** Statistically significant; # adjusted mean (standard error);* ***bold****= clinically significant (=/> 2 point reduction in total 6GSI; see Methods -* Measurement Tools *section)*

***Abbreviations:*** *6GSI = 6-item Gastrointestinal Severity Index; CI = Confidence Intervals; COM = Combined Treatment Group (synbiotic + gut-directed hypnotherapy); MD = Mean Difference; SYN = Synbiotic Treatment Group; t = x weeks*
